# Optimum dose of vitamin D for disease prevention in older people: BEST-D trial of vitamin D in primary care

**DOI:** 10.1007/s00198-016-3833-y

**Published:** 2016-12-16

**Authors:** H. Hin, J. Tomson, C. Newman, R. Kurien, M. Lay, J. Cox, J. Sayer, M. Hill, J. Emberson, J. Armitage, R. Clarke

**Affiliations:** 1 Hightown Surgery, Banbury, Oxfordshire, UK; 2 Clinical Trial Service Unit (CTSU) and Epidemiological Studies Unit and MRC Population Health Research Unit, Nuffield Department of Population Health, University of Oxford, Oxford, UK; 3 Department of Medicine, Division of Endocrinology and Metabolism, New York University Langone Medical Center, New York, NY USA

**Keywords:** Clinical trial, Markers of vitamin D status, Optimum dose, Vitamin D

## Abstract

**Summary:**

This trial compared the effects of daily treatment with vitamin D or placebo for 1 year on blood tests of vitamin D status. The results demonstrated that daily 4000 IU vitamin D3 is required to achieve blood levels associated with lowest disease risks, and this dose should be tested in future trials for fracture prevention.

**Introduction:**

The aim of this trial was to assess the effects of daily supplementation with vitamin D3 4000 IU (100 μg), 2000 IU (50 μg) or placebo for 1 year on biochemical markers of vitamin D status in preparation for a large trial for prevention of fractures and other outcomes.

**Methods:**

This is a randomized placebo-controlled trial in 305 community-dwelling people aged 65 years or older in Oxfordshire, UK. Outcomes included biochemical markers of vitamin D status (plasma 25-hydroxy-vitamin D [25[OH]D], parathyroid hormone [PTH], calcium and alkaline phosphatase), cardiovascular risk factors and tests of physical function.

**Results:**

Mean (SD) plasma 25(OH)D levels were 50 (18) nmol/L at baseline and increased to 137 (39), 102 (25) and 53 (16) nmol/L after 12 months in those allocated 4000 IU, 2000 IU or placebo, respectively (with 88%, 70% and 1% of these groups achieving the pre-specified level of >90 nmol/L). Neither dose of vitamin D3 was associated with significant deviation outside the normal range of PTH or albumin-corrected calcium. The additional effect on 25(OH)D levels of 4000 versus 2000 IU was similar in all subgroups except for body mass index, for which the further increase was smaller in overweight and obese participants compared with normal-weight participants. Supplementation with vitamin D had no significant effects on cardiovascular risk factors or on measures of physical function.

**Conclusions:**

After accounting for average 70% compliance in long-term trials, doses of 4000 IU vitamin D3 daily may be required to achieve plasma 25(OH)D levels associated with lowest disease risk in observational studies.

**Electronic supplementary material:**

The online version of this article (doi:10.1007/s00198-016-3833-y) contains supplementary material, which is available to authorized users.

## Introduction

About half of all women and one fifth of all men aged 50 years or older will experience a fracture in their lifetime, due chiefly to underlying osteoporosis [1]. Osteoporosis is characterized by decreased bone mass and typically presents with fractures of the wrist, spine and hip [2]. Observational studies indicate that low plasma levels of 25-hydroxy-vitamin D (25[OH]D), a widely used marker of vitamin D status, are associated with higher risks of osteoporosis [3, 4] and fractures [5, 6], in addition to vascular and non-vascular mortality [6, 7]. While correction of severe vitamin D deficiency is protective against rickets in children [8] and osteomalacia in adults [9], there is substantial uncertainty about whether vitamin D supplementation in individuals with moderate vitamin D insufficiency can prevent osteoporotic fracture or other disease outcomes. Previous trials [10–17] and meta-analyses of trials [18–21], assessing the effects of equivalent daily doses of 400–800 IU (10–20 μg) of vitamin D on risk of fracture, have reported conflicting results. However, no large trials for disease prevention have used doses of vitamin D sufficient to achieve and maintain plasma levels of 25(OH)D associated with the lowest risks of mortality and fractures reported in observational studies [6, 7]. Moreover, a recent systematic review of vitamin D and multiple health outcomes concluded that there was no consensus on the optimal dose or plasma level of 25(OH)D for disease prevention [22]. Dose-finding trials are required to interpret the results of the ongoing trials and to design a large trial of higher doses of vitamin D for disease prevention in older people.

Observational studies suggest that the lowest risks of mortality and morbidity are associated with plasma 25(OH)D levels around 90 nmol/L (36 ng/mL) [23]; this is also the level typically found in young British adults at the end of the summer months [24] and in people living in high sun-exposed regions [25, 26] and the levels associated with the lowest risks of vascular and non-vascular mortality in older people [6, 7]. In addition, plasma levels of parathyroid hormone (PTH) are inversely associated with plasma levels of 25(OH)D up to levels of about 70–100 nmol/L [27–29]. Previous trials have demonstrated that the effects of supplementation with 400 IU (10 μg) of vitamin D would only increase plasma 25(OH)D levels by 7–10 nmol/L [28, 29], but with typical compliance observed in long-term trials of vitamin D for disease prevention [15], only about 70% of this effect might be seen. Since the average plasma 25(OH)D levels in older people in the UK are about 50 nmol/L in the summer months (and much lower in winter), daily doses of 400 to 800 IU would not be sufficient to achieve and maintain plasma 25(OH)D levels >90 nmol/L throughout the year [28, 29].

The aims of the Biochemical Efficacy and Safety Trial of vitamin D (BEST-D) were to compare the effects of daily supplementation with either 4000 IU vitamin D or 2000 IU vitamin D or placebo on biochemical markers of vitamin D status in order to determine the optimum dose to use in a large randomized trial for the prevention of fractures and other health outcomes in older people living in the UK. The trial also investigated the effects of vitamin D on cardiovascular risk factors and on clinical tests of physical function.

## Materials and methods

The study design and pre-specified data analysis plan for the BEST-D trial have been previously reported [30]. In summary, BEST-D is a double-blind, randomized placebo-controlled, parallel group trial comparing the effects of daily supplementation with either 4000 IU (100 μg) or 2000 IU (50 μg) cholecalciferol (vitamin D3) or placebo for 12 months on biochemical markers of vitamin D status, cardiovascular risk factors and clinical tests of physical function [29]. About 300 men and women aged 65 years or older were recruited from a single general practice in Banbury, Oxfordshire, UK. Individuals who were ambulatory, living in the community and not currently taking more than 400 IU (10 μg) vitamin D3 daily were eligible to participate. All participants provided written informed consent, and the study was approved by the National Research Ethics Service Committee (Oxford B), UK. All trial investigators, trial staff and participants were kept blinded to the treatment allocation until the trial had been completed, the data analysis plan had been finalized and the database had been locked for analysis.

### Randomization and interventions

A research nurse visited participants at home and recorded a history of vascular disease or presence of vascular risk factors, falls and fractures and daily dietary intake of calcium; assessed symptoms of muscle and joint pain (using a ten-point visual scale with 10 being the most severe); and measured height, weight, blood pressure, arterial stiffness and handgrip strength (average of 3 measures on each hand) with a Jamar dynamometer [31]. Blood pressure and arterial stiffness were also measured at each visit after 10-min rest in the seated position [32]. First, a finger probe (PulseTrace PCA 2) was placed on the right forefinger to record the digital volume pulse using photoplethysmography over 30–60 s. This was followed by recording the mean of two blood pressure and brachial artery arterial stiffness measurements (aortic pulse wave velocity [PWV] and aortic augmentation index) made over 2 min using a TensioClinicTM® Arteriograph [33]. Blood was collected into vacutainers containing either lithium heparin or ethylenediaminetetraacetic acid (EDTA) for analyses. Randomization to study treatment (vitamin D3 4000 or 2000 IU or placebo daily) was by telephone to the coordinating centre where a computer-based minimization algorithm was used to ensure matching of the allocated groups by age, body mass index [BMI], smoking history, ethnicity and history of fracture. Vitamin D3 and matching placebo soft gel capsules were provided by Tishcon Corporation (Westbury, NY, USA).

### Follow-up visits

All participants were visited again in their homes by the study nurse at 6 and 12 months after randomization. In addition, one third of the participants were randomly selected to have a blood sample collected at 1 month. At the 6- and 12-month visits, information was recorded on compliance, serious adverse events, non-serious adverse events leading to discontinuation of study treatment, symptoms of muscle and joint pain, measures of physical function (handgrip strength) [34, 35] and mood (using the geriatric depression four-point scale) [36, 37], and a blood sample was collected. The participants were asked to attend a single assessment centre at their local general practice after their 12-month assessment to have clinical tests of physical function (chair rises, tests of balance and a 3-m walk) [34, 35] and for bone density at the heel and wrist bones measured using an OsteoSys EXA-300 scanner (OsteoSys, Seoul, Korea).

### Biochemical analyses

Plasma levels of 25(OH)D were selected as the best measure of vitamin D status rather than 1,25-dihydroxy-vitamin D because of its long half-life and direct relationship to intake and synthesis. Plasma 25(OH)D levels and plasma PTH were measured using an Access 2 immunoassay analyzer (Beckman Coulter Ltd., High Wycombe, England). The laboratory participated in the international DEQAS scheme for 25(OH)D and had a mean (SD) bias of −11.8% (7.5) from the target value over the period of the study. The performance target for the scheme was ±25% of the target value, which is assigned by the NIST reference measurement procedure. Plasma levels of albumin, calcium, phosphate and alkaline phosphatase were measured using a UniCel DxC 800 Synchron clinical system (Beckman Coulter Ltd., High Wycombe, England), and plasma levels of high-sensitivity C-reactive protein were measured using a BN ProSpec system (Siemens, Frimley, England); all assays used the suppliers’ reagents and calibrators. Further details of assays and performance characteristics are recorded in the Online Resource.

### Statistical analyses

All efficacy and safety assessments were conducted according to the intention-to-treat principle and followed the pre-specified data analyses set out in the trial Statistical Analysis Plan [30]. The co-primary outcomes were mean plasma 25(OH)D levels and the percentage of participants with plasma 25(OH)D levels >90 nmol/L at 12 months. The primary assessment of these outcomes was to compare participants allocated 4000 IU with those allocated 2000 IU daily (with secondary assessments including comparisons of each dose with placebo). Tertiary assessments of the co-primary outcomes involved comparison of the two active doses of vitamin D in the following subgroups: sex, age (<70, ≥70 years), body mass index (BMI <25, ≥25 to <30, ≥30 kg/m^2^), plasma 25(OH)D level at baseline (≤50, >50 nmol/L), dietary calcium intake (≤700, >700 mg/day), estimated glomerular filtration rate (eGFR ≤75, >75 ml/min/1.73 m^2^) and history of cardiovascular disease (CVD). A range of secondary and tertiary outcomes were also pre-specified, including mean 25(OH)D and percentage of participants with 25(OH)D >90 nmol/L at 1 and 6 months; percentage of participants with PTH in the reference interval at 1, 6 and 12 months; percentage of participants with calcium above the reference interval at 1, 6 and 12 months; mean values of all other biochemical tests and measures of vascular function at 6 and 12 months; and physical function at 12 months. Comparisons of mean follow-up values between treatment arms involved analysis of covariance (ANCOVA) adjusted, where possible, for the baseline value (with multiple imputation used to impute the few missing data). ANCOVA provides a more powerful test of the null hypothesis than either a comparison of mean follow-up values in isolation or a comparison of mean changes from baseline [38]. Comparisons of dichotomous outcomes were done using standard methods for 2 × 2 contingency tables. All *p* values were two sided and considered statistically significant, without allowance for multiple testing, if they were <0.05 [39]. Analyses were conducted using SAS version 9.3 and R version 2.11.1. The study was designed to have >90% power (at 2*p*=0.01) to detect a true difference in mean 25(OH)D between the two active doses at 12 months of 11 nmol/L (assuming an SD of 20 nmol/L). In the two active dose arms, it also had >80% power at 2*p*=0.05 to detect an increase in the proportion achieving a 12-month concentration >90 nmol/L from 60% to 80%. All of the analyses were conducted independently of the sources of support.

### Results

**Fig. 1:**
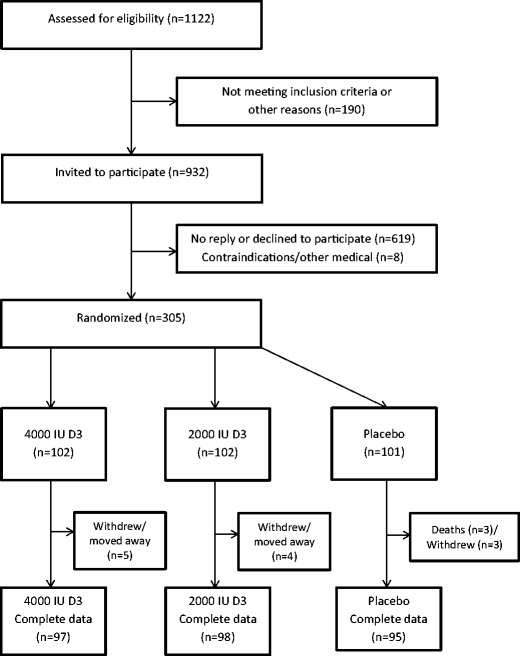
CONSORT flow diagram for the BEST-D trial

Figure 1 shows the outcome of the 1122 people who were assessed for eligibility for invitation to participate in the trial. Between 24 September 2012 and 14 March 2013, 305 participants were randomly allocated to take either 4000 IU D3 (*n* = 102), 2000 IU D3 (*n* = 102) or placebo (*n* = 101) daily.

### Baseline characteristics

The mean age was 72 years, 51% were men, and 7% were current smokers (Table 1). About 12% reported taking low-dose vitamin D3 (400 IU daily or less), and 3% reported using calcium supplements. While the self-reported physical activity ratings were high, one third of participants reported muscle aches and pains and two thirds reported joint aches and pains.

**Table 1:** Selected baseline characteristics, by allocated treatment

	4000 IU/day (*n* = 102)	2000 IU/day (*n* = 102)	Placebo (*n* = 101)
Age (years)	71 (6)	72 (6)	72 (6)
Male	52 (51%)	51 (50%)	52 (51%)
Current smoker	7 (7%)	7 (7%)	7 (7%)
Dietary calcium (mg/day)	724 (287)	695 (292)	713 (302)
Prior disease			
Heart disease^a^	20 (20%)	11 (11%)	11 (11%)
Stroke/TIA	5 (5%)	8 (8%)	6 (6%)
Hypertension	40 (39%)	44 (43%)	35 (35%)
Diabetes	9 (9%)	9 (9%)	9 (9%)
Fracture (ever)	31 (30%)	30 (29%)	30 (30%)
Any fall in the past 6 months	13 (13%)	15 (15%)	12 (12%)
Medication			
Any antihypertensive	50 (49%)	52 (51%)	46 (46%)
Statin	32 (31%)	29 (28%)	23 (23%)
Any antithrombotic	20 (20%)	23 (23%)	18 (18%)
Vitamin D (≤400 IU/day)	12 (12%)	10 (10%)	13 (13%)
Calcium	4 (4%)	1 (1%)	4 (4%)
Physical measurements			
Height (cm)	168 (10)	168 (10)	167 (10)
Weight (kg)	77 (17)	78 (15)	79 (15)
Body mass index (kg/m^2^)	27 (5)	27 (4)	28 (5)
Grip strength (kg)	25 (11)	25 (11)	25 (11)
Blood pressure and arterial stiffness			
Systolic blood pressure (mmHg)	133 (21)	132 (17)	129 (19)
Diastolic blood pressure (mmHg)	78 (11)	77 (10)	77 (12)
Heart rate (beats/min)	66 (10)	66 (12)	65 (9)
Pulse wave velocity (m/s)	10.0 (1.9)	9.6 (1.6)	9.7 (1.8)
Aortic augmentation index (%)	38 (16)	37 (14)	36 (15)
Pulse trace stiffness index (%)	9.2 (2.3)	9.1 (2.4)	9.5 (2.8)
Pulse trace reflection index (%)	64 (14)	63 (15)	67 (12)
Physical activity and muscle/joint pain			
Physical activity rating (1–10)	6.5 (2.0)	6.2 (2.0)	6.5 (2.0)
Any muscle aches/pains	43 (42%)	35 (34%)	38 (38%)
Any joint aches/pains	66 (65%)	66 (65%)	64 (63%)

Mean (SD) or % shown

^a^Defined as heart attack, angina or heart failure

### Compliance

Among those allocated to 4000 IU, 2000 IU or placebo, 93%, 93% and 87%, respectively, reported taking their capsules on all or most days at 6 months, while 90, 92 and 85% reported doing so at the 12-month visit. Overall, only 5%, 4% and 6% of those allocated to 4000 IU, 2000 IU or placebo were unable to attend their scheduled final visits (Fig. 1).

### Effects on plasma 25(OH)D levels

**Fig. 2:**
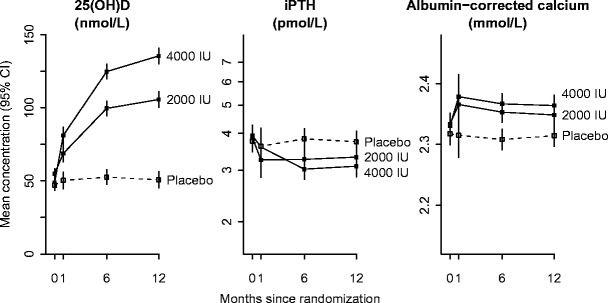
Effect of vitamin D allocation on mean plasma levels of 25(OH)D, intact parathyroid hormone and albumin-corrected calcium. The vertical axis represents about 8 baseline SDs for 25(OH)D and about 4 baseline SDs for iPTH and albumin-corrected calcium. Arithmetic means are shown for 25(OH)D and albumin-corrected calcium and geometric means for iPTH. The *p* values for tests of difference between the groups at each time point are given in Table 2

Mean (SD) plasma 25(OH)D levels were about 50 (18) nmol/L at baseline and increased to 137 (39), 102 (25) and 53 (16) nmol/L respectively, after 12 months of treatment among those allocated 4000 IU, 2000 IU or placebo, respectively (Fig. 2, Table 2), and 88%, 70% and 1%, respectively, achieved a 25(OH)D level >90 nmol/L at 12 months. Figure 2 shows the mean plasma levels of 25(OH)D when measured on three to four visits over 12 months. In the 100 participants evaluated at 1 month, mean plasma 25(OH)D levels were already significantly elevated (Fig. 2). By 6 months, 86% and 64% of participants allocated to 4000 and 2000 IU vitamin D, respectively, had plasma 25(OH)D >90 nmol/L (compared with 2% of placebo-allocated participants). Between 6 and 12 months, the 25(OH)D mean levels continued to increase by 11 nmol/L and 5 nmol/L, respectively.

**Table 2:** Effect of vitamin D on mean (SE) plasma levels of 25(OH)D, iPTH, albumin-corrected calcium and alkaline phosphatase

	4000 IU/day (*n* = 102)	2000 IU/day (*n* = 102)	Placebo (*n* = 101)	*p* value^a^	*p* value^b^	*p* value^c^
Plasma 25(OH)D (nmol/L)						
Baseline	49 (1.5)	55 (2.2)	47 (1.5)	–	–	–
1 month	81 (1.9)	69 (1.8)	49 (1.9)	<0.0001	<0.0001	<0.0001
6 months	126 (2.4)	97 (2.4)	55 (2.4)	<0.0001	<0.0001	<0.0001
12 months	137 (2.4)	102 (2.4)	53 (2.4)	<0.0001	<0.0001	<0.0001
Plasma iPTH (pmol/L)						
Baseline	3.93 (0.193)	3.86 (0.148)	3.76 (0.157)	–	–	–
6 months	2.98 (0.070)	3.25 (0.076)	3.91 (0.093)	<0.0001	<0.0001	0.0092
12 months	3.05 (0.080)	3.31 (0.087)	3.82 (0.100)	<0.0001	0.0001	0.0283
Plasma albumin-corrected calcium (mmol/L)						
Baseline	2.33 (0.012)	2.33 (0.008)	2.32 (0.007)	–	–	–
6 months	2.36 (0.006)	2.35 (0.006)	2.32 (0.006)	<0.0001	<0.0001	0.05
12 months	2.36 (0.006)	2.34 (0.006)	2.32 (0.006)	<0.0001	0.0106	0.0386
Plasma alkaline phosphatase (IU/L)						
Baseline	58 (1.7)	61 (1.6)	59 (1.9)	–	–	–
6 months	57 (0.7)	58 (0.7)	59 (0.7)	0.0247	0.13	0.46
12 months	60 (0.9)	60 (1.0)	60 (1.0)	0.99	1.00	0.98

Arithmetic mean (SE) shown for 25(OH)D and albumin-corrected calcium and geometric mean (approximate SE) shown for iPTH and alkaline phosphatase. Means (SE) at 1, 6 and 12 months are adjusted for the baseline values, with missing data imputed using multiple imputation. Mean iPTH and albumin-corrected calcium levels were not pre-specified outcomes

^a^ *p* value comparing 4000 IU daily versus placebo

^b^ *p* value comparing 2000 IU daily versus placebo

^c^ *p* value comparing 4000 versus 2000 IU daily

### Effects in pre-specified subgroups

**Fig. 3:**
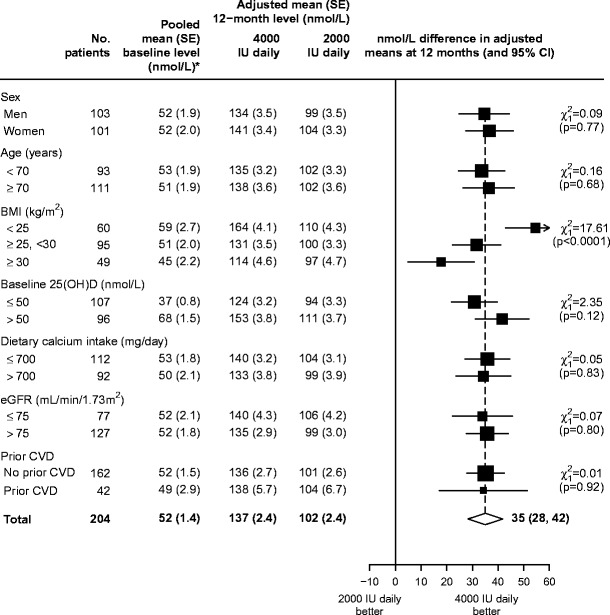
Additional effect on 12-month vitamin D level of 4000 IU daily compared with 2000 IU daily, overall and in pre-defined subgroups. Mean and SE estimates are adjusted for baseline 25(OH)D by analysis of covariance, with any missing values imputed through multiple imputation. The chi-square tests for trend shown for each subgroup test the null hypothesis of there being no linear trend in the difference in adjusted means across levels of the subgroup (as ordered) and are derived from standard formulae. The *white diamond* represents the overall estimated difference in baseline-adjusted 12-month mean 25(OH)D concentration between all patients allocated 4000 IU daily and all patients allocated 2000 IU daily. *Asterisk*: among patients allocated 4000 or 2000 IU daily

The effects of 4000 IU daily versus 2000 IU daily of vitamin D on plasma levels of 25(OH)D after 12 months were similar in all pre-specified subgroups, except when grouped by BMI, where the further increase from the higher dose on plasma 25(OH)D levels was smaller among those with higher baseline BMI (Fig. 3: *p* for trend <0.0001). The effects of 4000 versus 2000 IU dose of vitamin D on plasma levels of 25(OH)D were attenuated by one third in those who were overweight and by two thirds in those who were obese, compared with those with normal BMI. In a post hoc analysis of the effects of 2000 IU vitamin D versus placebo, however, the achieved difference in plasma 25(OH)D levels was similar in every subgroup, including by baseline BMI (Online Resource Fig. 1). The achieved differences in plasma levels of 25(OH)D at 6 and 12 months between those allocated 4000 versus 2000 IU daily and versus placebo were broadly similar when subdivided by the quartiles of plasma 25(OH)D levels at baseline (Online Resource Table 1).

### Effects on plasma PTH, calcium levels and alkaline phosphatase

Plasma levels of PTH at baseline were balanced by treatment allocation but were outside the laboratory normal range (1.1–6.8 pmol/L) in 22 participants. During follow-up, the proportion of participants with plasma PTH levels within the normal range was similar between those allocated 4000 IU daily, 2000 IU daily or placebo (respectively, 89%, 95% and 88% at 6 months and 91%, 95% and 87% at 12 months). Compared with placebo, mean PTH decreased significantly (*p* < 0.0001) with both doses of vitamin D (Fig. 2, Table 2), with lower mean plasma PTH levels at 6 and 12 months among participants allocated 4000 IU compared with participants allocated 2000 IU daily (*p* = 0.01 and *p* = 0.03, respectively). The reduction from baseline in plasma PTH levels at 12 months was 22% in the 4000 IU group and 14% in the 2000 IU group. Mean plasma levels of albumin-corrected calcium were also significantly increased by both vitamin D3 doses (Fig. 2, Table 2), but the absolute increases were small and not clinically apparent. The differences in 6- and 12-month plasma levels of albumin-corrected calcium between participants allocated 4000 IU daily and those allocated 2000 IU daily were also small (Table 2) and did not vary by baseline plasma levels of albumin-corrected calcium (Online Resource Table 2). Likewise, vitamin D3 had no significant effect on mean plasma levels of alkaline phosphatase at 12 months (Table 2). In post hoc analyses, there was no evidence that baseline plasma levels of plasma 25(OH)D modified the effect of allocation to vitamin D on 12-month alkaline phosphatase levels (*p* for interaction 0.89).

### Effects on cardiovascular risk factors and clinical tests of physical function

Table 3 shows the effects of vitamin D3 (both doses combined) on cardiovascular risk factors and on the physical function measurements at 12 months; Online Resource Table 3 gives the estimates at 6 months. Allocation to either dose of vitamin D3 had no significant effect on the physical measurements or measures of arterial stiffness at 12 months or on 12-month levels of total or LDL cholesterol, triglycerides, apolipoprotein B, NT-proBNP, phosphate, creatinine or urinary albumin/creatinine ratio (Table 3). Mean plasma levels of HDL cholesterol, apolipoprotein A_1_, C-reactive protein and albumin at 12 months were all slightly, but significantly, lower among participants allocated vitamin D3 (without allowing for multiple testing).

**Table 3:** Effect of allocation to 4000 or 2000 IU daily versus placebo on cardiovascular risk factors and self-reported and objective measures of physical function recorded at 12 months

Mean (SE) or *n* (%)	Either dose (*n* = 204)	Placebo (*n* = 101)	*p* value
Cardiovascular risk factors^a^			
Weight (kg)	77.8 (0.27)	78.4 (0.37)	0.17
Height (cm)	167.2 (0.15)	167.4 (0.21)	0.49
BMI (kg/m^2^)	27.7 (0.11)	27.9 (0.15)	0.24
Blood pressure and arterial stiffness			
Systolic blood pressure (mmHg)	132.2 (1.04)	131.8 (1.51)	0.82
Diastolic blood pressure (mmHg)	77.1 (0.65)	76.6 (0.96)	0.64
Heart rate (beats/min)	66.2 (0.60)	67.0 (0.87)	0.45
Pulse wave velocity (m/s)^b^	10.0 (0.10)	9.6 (0.14)	0.0223
Aortic augmentation index (%)	37.1 (0.93)	37.1 (1.38)	0.98
Pulse trace stiffness index (%)	9.4 (0.19)	9.5 (0.36)	0.96
Pulse trace reflection index (%)	67.2 (1.65)	66.3 (2.32)	0.77
Blood lipids and other blood biomarkers			
Total cholesterol (mmol/L)	5.24 (0.045)	5.29 (0.063)	0.51
LDL cholesterol (mmol/L)	2.84 (0.036)	2.83 (0.050)	0.90
HDL cholesterol (mmol/L)^b^	1.47 (0.011)	1.51 (0.016)	0.0180
Triglycerides (mmol/L)	1.71 (0.048)	1.66 (0.067)	0.58
Apolipoprotein A1 ( mg/dL)^b^	139 (0.7)	142 (1.0)	0.0250
Apolipoprotein B ( mg/dL)	94 (0.9)	94 (1.3)	0.95
Ln C-reactive protein (ln mg/dL)^b^	4.92 (0.005)	4.94 (0.007)	0.0208
Ln NT-proBNP (ln pg/mL)	6.11 (0.040)	6.23 (0.058)	0.11
Albumin (g/L)^b^	40.1 (0.13)	40.6 (0.20)	0.0222
Phosphate (g/L)	1.06 (0.009)	1.06 (0.013)	0.99
Ln creatinine (ln μmol/L)	4.35 (0.007)	4.35 (0.010)	0.54
Ln uACR (ln mg/mmol/L)	−0.26 (0.078)	−0.18 (0.119)	0.55
Self-reported physical function			
Any fracture	6 (3%)	1 (1%)	0.31
Any fall	34 (17%)	14 (14%)	0.53
Muscle pain			
Any pain	60 (29%)	26 (26%)	0.50
Severity (1–10)^c^	4.0 (0.28)	3.8 (0.46)	0.67
Joint pain			
Any pain	134 (66%)	60 (59%)	0.28
Severity (1–10)^c^	3.9 (0.20)	4.2 (0.27)	0.36
Physical activity rating (1–10)^a^	6.6 (0.09)	6.8 (0.12)	0.08
Geriatric depression score (0–4)^a^	2.1 (0.03)	2.1 (0.04)	0.27
Objective measures of physical function			
Grip strength (kg)^a^	23.8 (0.42)	24.4 (0.60)	0.40
Completion of 5 chair rises^d^	179 (88%)	82 (81%)	0.50
Balance in tandem position (completed 30 s)	123 (60%)	60 (59%)	0.88
Balance in semi-tandem position (completed 30 s)	178 (87%)	85 (84%)	0.46
Balance side by side (completed 30 s)	184 (90%)	87 (86%)	0.29
Failed to complete 30 s balance on left leg	139 (68%)	67 (66%)	0.70
Failed to complete 30 s balance on right leg	143 (70%)	64 (63%)	0.73
Completion of 3-m walk^d^	185 (91%)	87 (86%)	0.69
Bone density			
Wrist (T-score)	−2.1 (0.11)	−1.8 (0.14)	0.10
Heel (T-score)^b^	0.2 (0.12)	0.6 (0.15)	0.0308

^a^Missing data imputed using multiple imputation and adjusted, where available, for the baseline value

^b^This difference would not typically be considered statistically significant after allowance for multiple testing

^c^Among those with pain reported

^d^Time to completion (seconds) and percentage completion (%)

There were no significant differences in the reported clinical or adverse events with any vitamin D3 intake versus placebo on clinical events at 12 months or on self-reported fractures, falls, muscle pain severity, joint pain severity and physical activity ratings compared with those allocated to placebo (Table 3). Geriatric depression scores were also similar in both groups, as were the results of measures of physical function including handgrip strength, chair rises, balance and a 3-m walk. Moreover, the results of these clinical events and physical measures were similar at 6 months (Online Resource Table 3) and when the three treatment arms were considered separately (Online Resource Table 4). Bone density T-scores at the heel and wrist after 12 months were also broadly similar between the two groups (the *p* value of 0.03 for a lower heel T-score associated with allocation to vitamin D3 would not be statistically significant if allowance was made for multiple testing and levels at baseline were not measured to allow for any baseline imbalance to be assessed).

### Safety and tolerability

At randomization, albumin-corrected calcium was mildly elevated (>2.55 mmol/L) in eight participants (six who were subsequently allocated vitamin D3 and two who were subsequently allocated placebo). By 12 months, there were no new cases with elevated plasma levels of albumin-corrected calcium. Among the eight participants who had marginally elevated plasma levels of albumin-corrected calcium at baseline, six (five allocated vitamin D3 and one allocated placebo) still had elevated levels at 12 months, but none was considered clinically significant. Mean plasma levels of creatinine and phosphate in each of the treatment groups at 6 and 12 months were similar and similar to baseline values (data not shown).

At least one serious adverse event (SAE) was reported by 29 participants allocated 4000 IU, 30 allocated 2000 IU of vitamin D and 25 allocated placebo (Online Resource Table 5), respectively, and included three deaths (all deaths were among those allocated to placebo). None of the SAEs was considered treatment-related events. Study treatment tolerability was good and was discontinued before the scheduled end of the study by 17 participants (5 allocated 4000 IU, 5 allocated 2000 IU and 7 allocated placebo). A SAE was attributed as the reason for discontinuation in 3 participants (one in each group), and a non-SAE was the reason for discontinuation in 4 participants (1 allocated 4000 IU and 3 allocated placebo).

## Discussion

The present study demonstrates that supplementation with 4000 IU daily vitamin D3 compared with 2000 IU daily was associated with a significantly higher proportion of individuals achieving plasma levels of 25(OH)D >90 nmol/L (88% vs 70%, respectively) after 1 year of treatment. Mean plasma levels of PTH decreased significantly in both active vitamin D groups but were significantly lower in those allocated 4000 IU vitamin D daily compared to 2000 IU at both 6 and 12 months (albeit the magnitude of these differences was small). The intake of vitamin D at these high doses was well tolerated and was not associated with any adverse clinical events at 1 year. No participant developed clinical hypercalcaemia or kidney stones. Supplementation with these high doses of vitamin D had no detectable effects on cardiovascular risk factors or on measures of physical function after 1 year of treatment. The effects on biochemical markers of vitamin D status were consistent with previous studies of the safety of daily doses of 4000 IU daily vitamin D3 [37–39] and with studies indicating increases in plasma 25(OH)D levels of only about 7–10 nmol/L per 400 IU daily vitamin D3 [40–43]. The results of the present trial are also consistent with those of previous trials indicating significant differences in plasma 25(OH)D levels between daily doses of 2000 and 4000 IU [40]. Importantly, the trial demonstrated no adverse effects of higher doses of vitamin D on any of the biochemical parameters studied.

Previous randomized trials assessing vitamin D for fracture prevention typically tested daily doses of 400 to 800 IU (or equivalent doses administered intermittently) and so, even with good compliance, would only have increased 25(OH)D by at most 7–15 nmol/L. Observational studies indicate that a 10 nmol/L higher 25(OH)D is associated with 5–6% lower risk of any fracture and about 10% lower risk of hip fracture [6]. Even if half of this effect is reversible in a trial of about 5 years, to detect such modest effects would have required very large long-term randomized studies with these low doses of vitamin D. Consequently, the results of previous trials and meta-analyses of such trials for the prevention of fractures have been conflicting [12–16, 18, 19, 21]. Overall, previous trials of vitamin D when administered alone in doses up to the equivalent of about 1100 IU daily (without supplemental calcium) have not demonstrated any significant effects on hip or non-vertebral fractures [16, 18, 19]. The findings of the present trial suggest that these early trials assessed doses of vitamin D, which were an order of magnitude too low, and included too few people at increased fracture risk to have sufficient power to detect plausible effects on fracture.

Several large randomized trials are currently testing the effects of vitamin D at various higher doses on cardiovascular and other outcomes: 2000 IU daily (VITAL [*n* = 25,897]) [44]; 60,000 IU monthly (D-Health [*n* = 20,000]) [44]; 100,000 IU monthly (ViDA [*n* = 5100]); and 60,000 IU monthly (TIPS3 [*n* = 5500]); further smaller studies [45, 46] are testing other doses. However, even in combination, these trials are unlikely to have sufficient numbers of participants with incident fractures to clearly demonstrate plausible effects on osteoporotic fractures. Furthermore, large intermittent doses of vitamin D are associated with higher risks of fracture and falls, while daily doses may not be. Further large randomized trials, assessing doses of at least 4000 IU daily on risk of fracture in high-risk populations, are now required to address this question.

Some observational studies have suggested that plasma levels of 25(OH)D above 125 nmol/L may be harmful [47], but such studies cannot exclude reverse causality. The Institute of Medicine (USA) Committee recommended a dietary allowance of 600 IU/day and a daily upper-level intake of vitamin D of 4000 IU [42]. Moreover, their recent report did not identify any safety concerns with supplementation with 4000 IU/day over a 1-year period [48]. Nevertheless, the long-term safety and effectiveness for fracture prevention of such high doses (and the relevance of high plasma levels of 25[OH]D) can only be reliably tested in large long-term randomized trials.

Based on currently available evidence, the Scientific Advisory Committee on Nutrition in the UK advocated a reference nutrient intake of 400 IU vitamin D daily for the general population aged over 4 years and an upper limit of 4000 IU/day [43]. Hence, the doses recommended for assessing in a future large trial by the present study are substantially larger than the reference nutrient intake and are close to the recommended upper limit for both the USA and the UK populations. However, the BEST-D trial reported no adverse effects of 4000 IU/day on hypercalcaemia or any other of the biochemical variables studied.

The chief strengths of the present study are that it randomized a substantial number of healthy older people who are the group most at risk of osteoporotic fractures and demonstrated both acceptability and detailed biochemical safety of these doses over a 1-year period. Furthermore, both these doses eliminated the significant seasonal fall in plasma 25(OH) levels typically observed in this population (data not shown). The chief limitation of the present study is that almost all the study participants were Caucasian, so it cannot address any differential effects of vitamin D by ethnic group. A further limitation of the trial was that it was unable to address the effects of vitamin D on free 25(OH)D.

Several of the on-going trials are assessing the effects of vitamin D on cardiovascular events and all-cause mortality as their primary outcomes. The mechanisms underlying the associations between 25(OH)D and vascular and non-vascular mortality in the observational studies are uncertain, but the lack of any effects of these two high doses of vitamin D on the measured cardiovascular risk factors in the present trial does not support supplementation with vitamin D for prevention of CVD being mediated through changes in blood pressure, arterial stiffness, blood lipids or markers of inflammation. Nevertheless, this study cannot exclude any benefits for CVD prevention beyond 1 year of treatment.

Osteoporosis is a major public health problem that is associated with a high burden of fractures, and compelling indirect evidence implicates chronic vitamin D insufficiency as an important contributory cause. But previous trials of vitamin D have failed to reliably test the vitamin D hypothesis of osteoporosis because the doses of vitamin D tested in such trials were too low. After taking account of the typical average 70% compliance observed in long-term trials of vitamin supplements for disease prevention [15, 49], the results of the present study suggest that daily doses of 4000 IU of vitamin D3 may be required to achieve the high plasma levels of 25(OH)D associated with the lowest risks of mortality in the observational studies. However, trials testing the effects of such high doses of vitamin D should be conducted first before making recommendations on vitamin D supplementation for disease prevention in older people.

## Electronic supplementary material

ESM 1 (PDF 528 kb)
