# Supplementary material for: Optimum dose of vitamin D for disease prevention in older people: BEST-D trial of vitamin D in primary care
Source: Osteoporos Int. 2016 Dec 16;28(3):841–51. doi: 10.1007/s00198-016-3833-y (PMC5306173; doi:10.1007/s00198-016-3833-y)
Supplement: Supplementary file 1 — (PDF 528 kb) [file 198_2016_3833_MOESM1_ESM.pdf]

Online Resource material: Optimum dose of vitamin D for disease prevention in older people: BEST-D trial of vitamin D in primary care

Online Resource Methods **Pg**

1. Randomization 2
2. Primary and secondary outcomes 2
3. Laboratory measures 2

Online Resource Tables

1. Effect of allocation to 4000 versus 2000 IU daily vitamin D on plasma 25(OH)D at 6 and 12 months, by baseline 25(OH)D groups defined retrospectively 4
2. Effect of allocation to 4000 versus 2000 IU daily vitamin D on plasma albumin-corrected calcium at 6 and 12 months, by baseline albumin-corrected calcium groups defined retrospectively 5
3. Effects of allocation to 4000 or 2000 IU daily versus placebo on cardiovascular risk factors at 6 months 6
4. Pre-specified secondary outcomes in each randomized group 7
5. Serious adverse events, overall and by MedDRA system organ class

Online Resource Figures

1. Effect on 12−month vitamin D level of 2000 IU daily compared with placebo, overall and in pre−defined subgroups 9

Online Resource Methods

Randomization

Potentially eligible individuals were identified from a single general practice and were mailed a letter by their family doctor, together with a study information leaflet, to invite them to participate in the trial. Eligible individuals who agreed to participate in the trial were subsequently visited in their homes by a specially trained research nurse. After confirming eligibility and obtaining written informed consent, individuals were randomized using a central telephone randomization service. Allocation to study treatment (vitamin D3 4000 IU or 2000 IU or placebo daily) used a minimization algorithm that included age, body mass index [BMI], smoking history, ethnicity and history of fracture. Vitamin D3 and matching placebo administered in soft gel capsules were provided by Tischcon Corporation (Westbury, New York, USA).Participants were supplied with a six-month supply of study medication and were asked to take two capsules of either active vitamin D3 2000 IU, or matching placebo capsules daily.

Echocardiography to assess left ventricular function was undertaken on a subset of 150 participants at 12 months and these results will be reported separately.

Primary and secondary outcomes

The co-primary outcomes were mean plasma 25(OH)D levels and percentage of participants with 25(OH)D levels >90 nmol/L at 12 months. Secondary outcomes included: mean plasma 25(OH)D levels, and percentage of participants with 25(OH)D >90 nmol/L (36 ng/mL), at 1 and 6 months; percentage of participants with PTH in the normal range (1.1-6.8 pmol/L) at 1, 6 and 12 months; percentage of participants with albumin-corrected calcium levels above the normal range (2.15- 2.55 mmol/L) at 1, 6 and 12 months; mean level at 6 and 12 months of albumin, phosphate, creatinine, alkaline phosphatase and lipids; and blood pressure recorded at 6 and 12 months. Additional secondary outcomes included heart rate, blood pressure and brachial and digital arterial stiffness in all participants at 6 and 12 months. Tertiary outcomes assessed at 12 months included all site and specific fractures, falls, muscle pain, joint pain, self-assessed physical activity, number of respiratory infections, geriatric depression score, weight, height, BMI, hand grip strength, physical performance measures, and bone density T- and Z-scores at the hand and wrist. Safety outcomes included all serious adverse events, irrespective of whether these were considered to be related to study treatment, reasons for stopping study treatment and biochemical safety data.

Laboratory measures

Plasma levels of 25(OH)D and parathyroid hormone (PTH) were measured on blood samples collected using lithium heparin tubes on an Access 2 Immunoassay System (Beckman Coulter (UK) Ltd, High Wycombe, England). The 25(OH)D assay used a two-step competitive binding immunoenzymatic method traceable to the National Institute of Standards and Technology (Gaithersberg, Maryland USA) standard reference material (NIST SRM) 2972 and the PTH assay used a two-site immunoenzymatic sandwich method traceable to the World Health Organisation International Standard 79/500. The between-run precision for 25(OH)D levels was 6.0% at a level of 30.1 ng/mL and 8.7% at a level of 16.7 ng/mL and for PTH was 3.2% at a level of 146.5 pg/mL and 7.7% at a level of 19.7 pg/mL. To convert plasma 25(OH)D levels from nmol/L to ng/mL, divide by 2.5. To convert PTH from pmol/L to pg/ml divide by 0.11.

Using a UniCel DxC 800 Synchron Clinical System (Beckman Coulter (UK) Ltd, High Wycombe, England) and lithium heparin plasma, end point absorbance methods were used to measure plasma levels of albumin (assay traceable to NIST 927a), calcium (assay traceable to NIST 956) and phosphate (assay traceable to NIST 3139a). A kinetic rate absorbance method was used to measure alkaline phosphatase. Calibration of the alkaline phosphatase procedure was based on a bichromatic extinction coefficient for p-Nitrophenol, which has

a molar absorptivity of 17,900 at 410/480 nm. Between-run precision for albumin was 1.4% at a level of 31.1 g/L and 1.6% at a level of 6.2 g/L; for calcium 1.2% at levels of 2.49 mmol/L and 3.28 mmol/L, for phosphate 1.9% at levels of 1.37 mmol/L and 2.20 mmol/L and for alkaline phosphate 7.2% at a level of 23.0 IU/L and 3.7% at a level of 55.3 IU/L. Creatinine and albumin concentrations in urine were also assayed on the UniCel DxC 800 Synchron Clinical System using a modified Jaffe rate method and turbidimetric method, respectively, with the recommended manufacturers' reagents, calibrators and settings. Albuminuria was calculated from the urine creatinine and urine microalbumin ratio. Between-run precision for urine creatinine was 1.0% at a level of 17.3 mmol/L and 1.4% at a level of 7.15 mmol/L and for urine microalbumin 2.5% at levels of 183.0 mg/L and 5.0% at levels of 25.6 mg/L.

Using an AU680 Chemistry System (Beckman Coulter (UK) Ltd, High Wycombe, England) and EDTA plasma, end point absorbance methods were used to measure plasma levels of cholesterol (assay traceable to NIST 909b), LDL cholesterol (assay traceable to the CDC LDL cholesterol reference method), HDL cholesterol (assay traceable to the CDC HDL cholesterol reference method) and triglycerides (assay traceable to the Isotope Dilution Mass Spectrometry Reference Method); and turbidimetric methods were used to measure plasma levels of apolipoprotein A1 (assay traceable to the WHO – IFCC International Reference Material SP1-01 and SP-03), apolipoprotein B (assay traceable to the WHO – IFCC International Reference Material SP3-07). The LDL cholesterol and HDL cholesterol methods used were the Genzyme direct methods manufactured by Sekisui Medical, Tokyo. The CTSU laboratories are accredited (ISO 17025:2005) by the UK Accreditation service for the lipid assays described above. Between-run precision for cholesterol was 1.5% at a level of 7.66 mmol/L and 1.3% at a level of 3.53 mmol/L; for LDL cholesterol 2.1% at levels of 3.63 mmol/L and 1.73 mmol/L, for HDL cholesterol 3.1% at a level of 2.88 mmol/L and 2.9% at a level of 1.01 mmol/L; for triglycerides 1.7% at a level of 2.93 mmol/L and 1.6% at a level of 1.11 mmol/L; for apolipoprotein B 1.9% at a level of 135.2 mg/dL and 1.7% at a level of 69.0 mg/dL; and for apolipoprotein A1 1.5% at a level of 124.1 mg/dL and 2.0% at a level of 61.2 mg/dL.

Plasma high sensitivity C-reactive protein levels were measured in EDTA plasma on a Siemens BN ProSpec System using an immunoassay method (assay calibrated with reference to ERM-DA470). Between-run precision for high sensitivity C-reactive protein was 3.4% at a level of 9.63 mg/L and 3.1% at a level of 1.22 mg/L.

References

Clarke R, Newman C, Tomson J, et al. Estimation of the optimum dose of vitamin D for disease prevention in older people: Rationale, design and baseline characteristics of the BEST-D trial. *Maturitas* 2015;80: 426-431.

Online Resource Table 1: Effect of allocation to 4000 versus 2000 IU daily vitamin D on plasma 25(OH)D at 6 and 12 months, by baseline 25(OH)D groups defined retrospectively

	6 months			12 months		
**Baseline 25(OH)D, nmol/L**	**4000 IU daily**	**2000 IU daily**	**Difference**	**4000 IU daily**	**2000 IU daily**	**Difference**
<36	115 (5.0)	89 (4.6)	26 (6.8)	123 (5.0)	91 (4.5)	32 (6.7)
36 to <48	112 (4.0)	93 (4.8)	20 (6.3)	120 (3.9)	98 (4.6)	22 (6.1)
48 to <60	132 (5.0)	92 (5.9)	40 (7.7)	141 (5.1)	103 (5.9)	38 (7.8)
60+	145 (4.9)	111 (4.0)	34 (6.3)	167 (5.6)	116 (4.6)	50 (7.3)

Arithmetic mean (SE) shown. Means and SEs are adjusted for baseline values, with missing data imputed using multiple imputation. Four baseline groups defined by the quartiles of the distribution in all participants.

Online Resource Table 2: Effect of allocation to 4000 versus 2000 IU daily vitamin D on plasma albumin-corrected calcium at 6 and 12 months, by baseline albumin-corrected calcium groups defined retrospectively

Baseline albumin-corrected calcium, mmol/L	6 months			12 months		
	4000 IU daily	2000 IU daily	Difference	4000 IU daily	2000 IU daily	Difference
<2.28	2.28 (0.011)	2.29 (0.011)	-0.01 (0.016)	2.30 (0.012)	2.28 (0.013)	0.02 (0.017)
2.28 to <2.32	2.33 (0.012)	2.32 (0.010)	0.01 (0.016)	2.32 (0.012)	2.32 (0.011)	0.00 (0.016)
2.32 to <2.37	2.37 (0.011)	2.35 (0.011)	0.02 (0.016)	2.37 (0.011)	2.35 (0.012)	0.02 (0.016)
2.37+	2.45 (0.010)	2.44 (0.009)	0.01 (0.013)	2.44 (0.012)	2.43 (0.011)	0.00 (0.016)

Arithmetic mean (SE) shown. Means and SEs are adjusted for baseline values, with missing data imputed using multiple imputation. Four baseline groups defined by the quartiles of the distribution in all participants.

Online Resource Table 3: Effects of allocation to 4000 or 2000 IU daily versus placebo on cardiovascular risk factors at 6 months

	Either dose (n=204)	Placebo (n=101)	P
Blood pressure and arterial stiffness			
Systolic blood pressure, mmHg	129.8 (0.94)	127.8 (1.44)	0.25
Diastolic blood pressure, mmHg	76.2 (0.66)	75.5 (1.02)	0.60
Heart rate, beats/min	65.1 (0.59)	66.7 (0.92)	0.14
Pulse wave velocity, m/s	9.8 (0.09)	9.9 (0.13)	0.61
Aortic augmentation index, %	37.1 (0.96)	35.0 (1.33)	0.20
Pulse trace stiffness index, %	9.6 (0.17)	9.5 (0.24)	0.74
Pulse trace reflection index, %	65.0 (0.96)	65.2 (1.34)	0.93
Blood lipids and other blood biomarkers			
Total cholesterol, mmol/L	5.20 (0.044)	5.10 (0.062)	0.18
LDL cholesterol, mmol/L	2.84 (0.032)	2.76 (0.045)	0.13
HDL cholesterol, mmol/L	1.44 (0.011)	1.45 (0.016)	0.41
Triglycerides, mmol/L	1.68 (0.046)	1.61 (0.066)	0.41
Apolipoprotein A1, mg/dL	137 (0.6)	138 (0.9)	0.42
Apolipoprotein B, mg/dL	94 (0.9)	92 (1.2)	0.18
Ln C-reactive protein, ln mg/dL	4.91 (0.005)	4.92 (0.007)	0.34
Albumin, g/L	39.7 (0.14)	39.9 (0.20)	0.36
Phosphate, g/L	1.06 (0.010)	1.06 (0.014)	0.97
Ln creatinine, ln umol/L	4.36 (0.007)	4.34 (0.010)	0.19

Arithmetic mean (SE) shown. Means and SEs are adjusted for baseline values, with missing data imputed using multiple imputation.

Online Resource Table 4: Pre-specified secondary outcomes in each randomized group

	4000 IU/day (n=102)	2000 IU/day (n=102)	Placebo (n=101)	P[1]	P[2]	P[3]
1 month visit[4]						
Plasma 25(OH)D >90 nmol/L	9 (26%)	3 (9%)	1 (3%)	0.0246	0.35	0.06
iPTH within range 1.1-6.8 pmol/L	28 (82%)	34 (97%)	31 (94%)	0.16	0.53	0.07
Albumin-corrected calcium >2.55 mmol/L	3 (9%)	0 (0%)	0 (0%)	0.25	1.00	0.23
6 month visit						
Plasma 25(OH)D >90 nmol/L	88 (86%)	65 (64%)	2 (2%)	<0.0001	<0.0001	0.0003
iPTH within range 1.1-6.8 pmol/L	91 (89%)	97 (95%)	89 (88%)	0.81	0.08	0.13
Albumin-corrected calcium >2.55 mmol/L	5 (5%)	1 (1%)	0 (0%)	0.07	1.00	0.14
Albumin (g/L)	39.7 (0.20)	39.7 (0.20)	39.9 (0.20)	0.47	0.40	0.91
Phosphate (g/L)	1.08 (0.014)	1.04 (0.014)	1.06 (0.014)	0.38	0.42	0.09
Ln creatinine (ln umol/L)	4.36 (0.010)	4.35 (0.010)	4.34 (0.010)	0.15	0.41	0.54
Total cholesterol (mmol/L)	5.19 (0.061)	5.21 (0.062)	5.10 (0.062)	0.28	0.22	0.90
LDL cholesterol (mmol/L)	2.83 (0.045)	2.85 (0.045)	2.76 (0.045)	0.24	0.15	0.79
HDL cholesterol (mmol/L)	1.43 (0.015)	1.45 (0.016)	1.45 (0.016)	0.24	0.80	0.35
Triglycerides (mmol/L)	1.72 (0.064)	1.64 (0.066)	1.61 (0.066)	0.24	0.81	0.36
Apolipoprotein A1 (mg/dL)	136 (0.9)	138 (0.9)	138 (0.9)	0.19	0.95	0.21
Apolipoprotein B (mg/dL)	94 (1.2)	94 (1.2)	92 (1.2)	0.33	0.19	0.73
Ln hsCRP (ln mg/dL)	4.90 (0.007)	4.91 (0.007)	4.92 (0.007)	0.16	0.82	0.23
Systolic blood pressure (mm Hg)	129.7 (1.31)	129.9 (1.36)	127.8 (1.44)	0.34	0.29	0.90
Diastolic blood pressure (mm Hg)	75.9 (0.91)	76.5 (0.95)	75.5 (1.02)	0.81	0.49	0.63
Heart rate (beats/min)	64.9 (0.81)	65.3 (0.85)	66.7 (0.92)	0.14	0.27	0.71
Pulse wave velocity (m/s)	9.8 (0.13)	9.9 (0.13)	9.9 (0.13)	0.46	0.89	0.55
Aortic augmentation index (%)	36.7 (1.34)	37.5 (1.38)	35.0 (1.33)	0.36	0.19	0.68
Pulse trace stiffness index (m/s)	9.6 (0.25)	9.6 (0.24)	9.5 (0.24)	0.70	0.85	0.83
Pulse trace reflection index (m/s)	64.6 (1.40)	65.4 (1.31)	65.2 (1.34)	0.75	0.89	0.66
12 month visit						
iPTH within range 1.1-6.8 pmol/L	93 (91%)	97 (95%)	88 (87%)	0.36	0.05	0.27
Albumin-corrected calcium >2.55 mmol/L	4 (4%)	1 (1%)	1 (1%)	0.21	0.99	0.21
Albumin (g/L)	40.0 (0.19)	40.1 (0.19)	40.6 (0.20)	0.0370	0.06	0.84
Phosphate (g/L)	1.06 (0.013)	1.05 (0.013)	1.06 (0.013)	0.71	0.72	0.46
Ln creatinine (ln umol/L)	4.36 (0.010)	4.34 (0.010)	4.35 (0.010)	0.21	0.84	0.15
Total cholesterol (mmol/L)	5.26 (0.063)	5.22 (0.064)	5.29 (0.063)	0.70	0.45	0.70
LDL cholesterol (mmol/L)	2.85 (0.050)	2.84 (0.051)	2.83 (0.050)	0.86	0.97	0.89
HDL cholesterol (mmol/L)	1.47 (0.016)	1.46 (0.016)	1.51 (0.016)	0.06	0.0278	0.74
Triglycerides (mmol/L)	1.70 (0.067)	1.72 (0.068)	1.66 (0.067)	0.71	0.55	0.82
Apolipoprotein A1 (mg/dL)	139 (1.0)	138 (1.0)	142 (1.0)	0.09	0.0296	0.62
Apolipoprotein B (mg/dL)	94 (1.3)	94 (1.3)	94 (1.3)	0.95	0.97	0.98
Ln hsCRP (ln mg/dL)	4.92 (0.007)	4.92 (0.007)	4.94 (0.007)	0.08	0.0259	0.63
Ln NT-proBNP (ln pg/mL)	6.17 (0.056)	6.04 (0.057)	6.23 (0.058)	0.52	0.0281	0.11
Systolic blood pressure (mm Hg)	132.5 (1.43)	131.8 (1.51)	131.8 (1.51)	0.71	0.98	0.73
Diastolic blood pressure (mm Hg)	77.2 (0.89)	77.0 (0.94)	76.6 (0.96)	0.65	0.73	0.92
Heart rate (beats/min)	66.0 (0.84)	66.4 (0.87)	67.0 (0.87)	0.40	0.64	0.72
Pulse wave velocity (m/s)	10.1 (0.13)	9.9 (0.14)	9.6 (0.14)	0.0088	0.20	0.19
Aortic augmentation index (%)	37.5 (1.28)	36.8 (1.36)	37.1 (1.38)	0.83	0.86	0.69
Pulse trace stiffness index (m/s)	9.4 (0.28)	9.4 (0.27)	9.5 (0.36)	0.94	0.98	0.96
Pulse trace reflection index (m/s)	66.3 (2.17)	68.4 (2.53)	66.3 (2.32)	0.98	0.54	0.52

No. (%) or mean (SE) shown. Means and SEs are adjusted for baseline values, with missing data imputed using multiple imputation.
[1] P-value comparing 4000 IU daily versus placebo.
[2] P-value comparing 2000 IU daily versus placebo.
[3] P-value comparing 4000 versus 2000 IU daily.
[4] Among those selected for the 1 month sample.

Online Resource Table 5: Serious adverse events, overall and by MedDRA system organ class

	4000 IU/day (n=102)	2000 IU/day (n=102)	Placebo (n=101)
Any serious adverse event			
Blood or lymphatic system disorder	0	1	0
Cardiac disorder	3	2	2
Eye disorder	1	1	2
Gastrointestinal disorder	1	2	3
General disorders and administration site conditions	1	1	1
Infection/infestation	10	5	6
Injury, poisoning or procedural complication	4	4	1
Investigations	0	2	1
Metabolism/nutrition disorder	3	1	1
Musculoskeletal or connective tissue disorder	4	4	3
Neoplasms benign, malignant or unspecified (inc cysts and polyps)	6	5	5
Nervous system disorder	0	5	1
Psychiatric disorder	1	0	3
Renal/urinary disorder	0	0	1
Respiratory, thoracic or mediastinal disorder	1	0	0
Skin and subcutaneous tissue disorder	1	1	0
Surgical/medical procedure	1	4	4
Vascular disorder	1	2	0
Subtotal: Any serious adverse event	29	30	25

Counts are of the number of patients experiencing at least one event of the given type. There were 3 deaths among placebo-allocated patients (1 gastrointestinal death and 2 neoplastic deaths).

Online Resource Figure 1: Effect on 12−month vitamin D level of 2000 IU daily compared with placebo, overall and in pre−defined subgroups

	No. patients	Pooled mean (SE) baseline level (nmol/L)*	Adjusted mean (SE) 12−month level (nmol/L): 2000 IU daily	Adjusted mean (SE) 12−month level (nmol/L): Placebo	nmol/L difference in adjusted means at 12 months (and 95% CI)	
Sex						
Men	103	52 (2.0)	99 (3.5)	56 (3.4)		χ^2_1=2.39
Women	100	49 (1.9)	104 (3.3)	51 (3.4)		(p=0.12)
Age (years)						
< 70	92	52 (2.0)	102 (3.3)	54 (3.2)		χ^2_1=0.05
≥ 70	111	50 (1.9)	102 (3.6)	53 (3.5)		(p=0.83)
BMI (kg/m^2)						
< 25	59	58 (2.5)	110 (4.3)	61 (4.3)		χ^2_1=0.02
≥ 25, <30	88	50 (2.2)	100 (3.3)	53 (3.6)		(p=0.89)
≥ 30	56	45 (2.2)	97 (4.7)	47 (4.0)		
Baseline 25(OH)D (nmol/L)						
≤ 50	110	37 (0.7)	94 (3.3)	41 (3.0)		χ^2_1=2.98
> 50	92	68 (1.6)	111 (3.7)	70 (4.0)		(p=0.08)
Dietary calcium intake (mg/day)						
≤ 700	114	51 (1.8)	104 (3.1)	53 (3.1)		χ^2_1=0.42
> 700	89	51 (2.1)	99 (3.9)	53 (3.9)		(p=0.52)
eGFR (mL/min/1.73m^2)						
≤ 75	74	50 (2.3)	106 (4.2)	51 (4.4)		χ^2_1=2.24
> 75	129	51 (1.7)	99 (3.0)	55 (2.8)		(p=0.13)
Prior CVD						
No prior CVD	170	51 (1.5)	101 (2.6)	54 (2.5)		χ^2_1=0.28
Prior CVD	33	49 (3.5)	104 (6.7)	51 (7.2)		(p=0.60)
**Total**	**203**	**51 (1.4)**	**102 (2.4)**	**53 (2.4)**	**48 (42, 55)**	

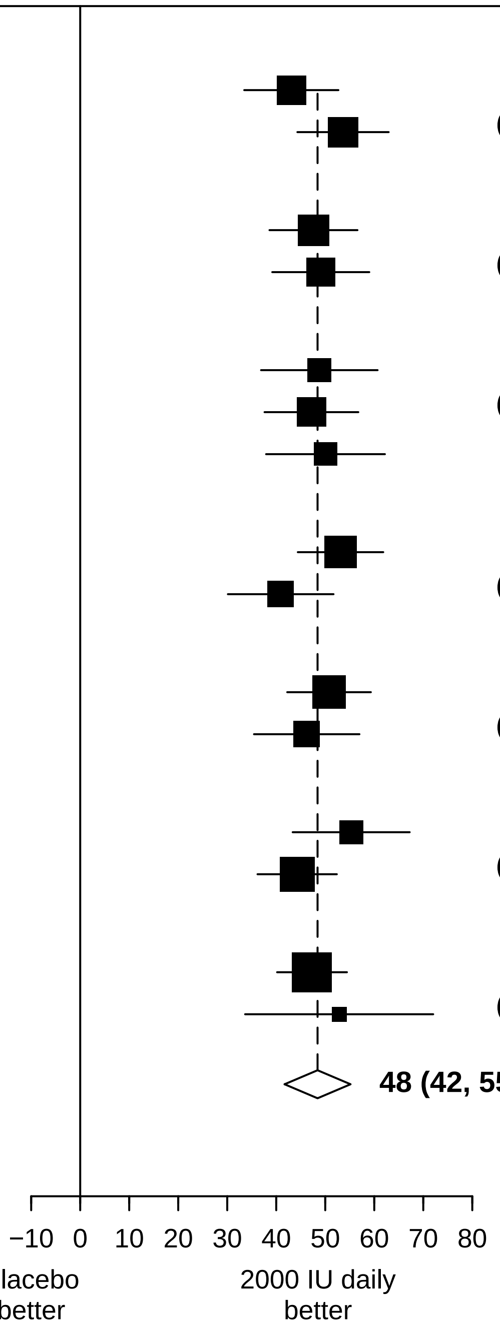

Mean and SE estimates are adjusted for baseline 25(OH)D by analysis of covariance, with any missing values imputed through multiple imputation. The chi−square tests for trend shown for each subgroup test the null hypothesis of there being no linear trend in the difference in adjusted means across levels of the subgroup (as ordered) and are derived from standard formulae. The white diamond represents the overall estimated difference in baseline−adjusted 12−month mean 25(OH)D concentration between all patients allocated 2000 IU daily and all patients allocated placebo.

* Among patients allocated 2000 IU daily or placebo.
